# Annotations

**Published:** 1893-10-07

**Authors:** 


### Annotations.

**The Late Professor Jowett.**

The late Professor Jowett was a typical Englishman though of a kind unhappily too rare. He was a clergyman without clericalism; a professor without pedantry; and although he was "a scholar, and a ripe and good one," his ambition first and above all things was to be an "English gentleman of simple manners." Of him the *Times* finely wrote that "Truthfulness and justice, the benevolence which springs from a constant and living sense of human brotherhood, and the moderation and modesty which have their root in self-respect, were amongst the qualities he most highly prized, while sincere and steady work was in his eyes at once the foundation and the test of moral progress." Work, sincere and steady work, the foundation and the test of moral progress. Here is a moral position which commends itself to the judgment of science. Probably science herself never defined more briefly or exactly the just and natural conditions of moral advancement. Yet Jowett was a clergyman, and a man of the "old learning." It would be an excellent thing if every leader of thought, and, indeed, every "sulphur match" striker of intellectual light, in newspapers, at universities, and elsewhere would possess himself of this fine, English, large and tolerant, but strong temper of Jowett's. We have a good deal of it in the medical profession. Our studies and our practical difficulties and failures make us both tolerant and patient. But we could do with more. The whole of our national, political and professional life would be a new thing if men of Jowett's mind and temper could be counted by thousands in every town.

**Huxley and Clericalism.**

Is Mr. Huxley quite just to the priestly element in human character? Hardly! That he has received scant measure of reasonableness at the hands of certain individual clerics we can readily admit. But will anybody deny that he has displayed an excellent capacity in returning a "Roland for an Oliver"? Nay, has he not sometimes administered the "Oliver" himself, and been compelled to receive a "Roland" in return? Mr. Huxley, in standing sponsor for an uniform edition of his own works, plants his feet by the old flag of defiance to "clericalism" in all its moods and tenses. "The objects I have had in view," says the professor, "are these . . . the popularisation of science . . . the development and organisation of scientific education . . . and untiring opposition to that ecclesiastical spirit, that clericalism, which, in England as everywhere else, and to whatever denomination it may belong, is the deadly enemy of science." Well, well, the old lion roars once more, and with a loud voice, but it was hardly necessary. The battle has been won, Mr. Huxley. You have converted the best of the clerics, and now they are on your own side. For the dull remainder it was hardly worth while to roar and lash your flanks. What is clericalism? It is human infallibility; infallibility in some man or some organisation of men, or some book. "There is no such infallibility," roars Lion Huxley. "We know it," answer High Church, Broad Church, and Low Church in united chorus. But there is religion, and there are morals, and we want authority for these; and when we have the unanimous consent of all sober thinking men, we feel we have authority enough. That consent, including Mr. Huxley's, is now gained. There is not a man anywhere whose opinion is worth a cheeseparing, who denies the value of good morals, or the necessity for a popular religion. Mr. Huxley's first considerable book was published in 1866. Concerning this, as of his other works, he tells us he has "nothing to alter in them." That is all very well from the point of view of a stubborn and determined consistency. But is it not possible that Mr. Huxley's sound opinions might be made more potent for the destruction of "clericalism," or, better still, for the improvement of the clerical mind, if the literary husks in which they are encased were a little less prickly and damaging to the mental fingers? Mr. Huxley is a born fighter; but it is pleasant to see even a fighter "put up his sword into its sheath," and smile genially; more especially when the sword is a sword of victory. If Mr. Huxley lives to see a new edition of his collected works called for, let us hope he will give us a little more of our Shakespeare's large mental placidity in his preface. A prose Dante is all very well; but in England a prose Shakespeare would be ten thousand times better.

**Dr. Bristowe and His Friends.**

Dr. Bristowe, the learned, the genial, and the inimitable, has been asked this week by his friends and colleagues at St. Thomas's Hospital to accept a portrait of himself, painted for them by an artist, Miss Bristowe, who is in the fullest sympathy with the object of those who are making the presentation. Dr. Bristowe has done much work of the first order for the profession of his choice, and has had many honours of the first class conferred upon him. But great as his work has been, and many as have been the honours of which he has been the recipient, it is the man himself, much more than his honours or his work, who lives enshrined in the tenderest part of the heart of every one who has been privileged to know him. In a short notice like the present, we can but give a brief catalogue of the most conspicuous circumstances of Dr. Bristowe's life. In 1850 he took the degree of M.B. London, and that of M.D. in 1852. In 1858 he became F.R.C.P. London. Dr. Bristowe is, or has been, president or fellow of most of the learned societies in London connected with the profession of medicine. He is also a Fellow of the Royal Society. For the full term allowed by the statutes he was physician to St. Thomas's Hospital, and is now consulting physician. His contributions to medical literature have been fairly numerous, and always of the first order of importance. Some of his literary work is monumental. Of this class may be mentioned "Clinical Lectures and Essays on Diseases of the Nervous System," "Lectures on Voice and Speech," and two octavo volumes on "The Theory and Practice of Medicine," which rank with the first works of their class throughout the civilised world. Dr. Bristowe, though now receiving what many people may consider the honours of a *Nunc Dimittis*, is by no means an old man, and we hope he will live for many years to serve the public with his ripe experience and to adorn the noble profession of which he is so universally beloved a member. This journal and those connected with it, as well as the Hospitals Association and the Royal National Pension Fund for Nurses, are under very special obligations to Dr. Bristowe, and all of us hold him in such esteem as is never given to any men but those who have been tried and proved in every conceivable way. Dr. Bristowe is much more than an ornament to medicine; he confers honour upon mankind. These are no mere words. If learning of the rarest, simplicity which is seldom paralleled, courage the most unyielding, a modesty which has rarely been equalled and never surpassed, combined with a goodness of heart so transparent as to be irresistible, entitle a man to honour of the widest kind, then our whole generation may uncover the head to Dr. Bristowe. The medical profession has not now, and has never known a more honourable and truly noble man.

